# Microplastics Induced Dysfunctions in Physiology and Behavior of Fish: A Comprehensive Review

**DOI:** 10.1155/jt/4669316

**Published:** 2026-04-06

**Authors:** Md. Asad Ud Zahan Siddique, Md. Sayem Sheikh, Md Shahjahan, Md Al-Emran

**Affiliations:** ^1^ Department of Fisheries Management, Laboratory of Fish Ecophysiology, Bangladesh Agricultural University, Mymensingh, 2202, Bangladesh, bau.edu.bd

**Keywords:** behavior, fish, microplastics, physiology, reproduction

## Abstract

Microplastics (MPs) pollution is a global concern due to their widespread persistence, occurrence, and toxicity to aquatic organisms. Fish are most vulnerable to MPs due to their feeding habits and ecological niches. This review critically synthesizes current evidences on MPs induced disruptions of key physiological functions and behavioral patterns in fish, with an emphasis on the underlying mechanistic pathways and biological consequences. MPs originate from various sources, entering aquatic systems and being ingested by fish directly or via trophic transfer. A prominent effect is growth inhibition commonly caused by gastrointestinal damage, impaired nutrient absorption, and metabolic stress. Moreover, it causes several hematobiochemical disruptions including anemia, leukocyte fluctuations, and biochemical and enzymatic imbalances that are connected to oxidative stress and immunosuppression. MPs also disrupt reproductive performances of fish through altering gonadosomatic index, inducing endocrine disruptions, dysregulating hypothalamic–pituitary–gonadal axis genes, and reducing fertilization and hatching success, with several transgenerational effects. Furthermore, MPs can induce oxidative stress through overproduction of reactive oxygen species (ROS) and modulation of antioxidant enzymes, along with dysregulation of immune and inflammatory responses. Behavioral alterations include reduced swimming performance and changes in feeding and reproductive behavior, which are linked to neurotoxicity and impairment of energy metabolism. Although a limited number of studies suggest species‐specific effects, most studies highlight significant adverse impacts on fish health. Hence, this review and meta‐analysis indicates that MPs substantially compromise fish physiology manifesting as poor growth, altered blood and metabolic profiles, impaired reproduction, and behavioral patterns. By integrating sources and transport pathways to physiological and behavioral outcomes, this review provides a comprehensive summary to inform ecological risk assessment and management of MPs in fisheries and aquatic ecosystems.

## 1. Introduction

Microplastics (MPs) are broadly defined as plastic particles with a diameter smaller than 5 mm. There are two major classes of MPs such as primary and secondary MPs. Primary MPs are intentionally manufactured at the microscale as microbeads in personal care products (PCPs) and industrial abrasives, whereas secondary MPs are smaller particles generated through fragmentation and weathering of larger plastic debris into micro‐sized particles [1, 2]. However, MPs also vary in physical features such as size, shape, color, density, etc. [3, 4] and in chemical composition including polyethylene (PE), polypropylene, polystyrene (PS), polyvinyl chloride (PVC), PE terephthalate, and so on [5, 6]. These physical and chemical properties regulate degradation time, environmental destiny, and toxicity levels [7–9].

**FIGURE 1:**
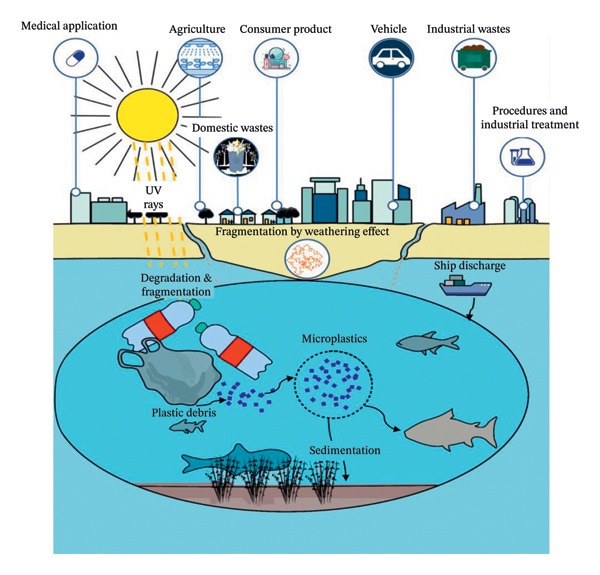
Sources of MPs in aquatic environment and possible transfer pathways to fish (figure is original and created by the authors of the manuscript).

In aquatic environments, MPs enter through several pathways, including urban runoff, wastewater effluents, industrial discharge, agriculture, aquaculture operations, chemical industries, and the breakdown of fishing gear and vessels [10–12]. Once these debris enter the environment, they become easily ingested by primary consumers at lower trophic levels, such as zooplankton, or by organisms at higher trophic levels including mollusks, crustaceans, and various types of fish [13–15]. Fish are particularly susceptible to the toxic effects of MPs due to their ecological niche, feeding behavior, and organismal physiology. As most MPs particles are of different colors, fish mistake them for food or ingest them indirectly through contaminated preys [16–18] (Figure 1).

The physiological effects of MPs exposure in fish are extensive and can adversely affect various physiological systems such as respiration, circulation, absorption, etc. The most prominent observable effect is growth hindrance. Moreover, physical injury to the gastrointestinal tract, reduction in feed intake, and impairment in nutrient absorption can be caused by MPs [19–21]. All these impairments lead to a reduction of weight gain (WG) and specific growth rate (SGR) and feed conversion efficiency in fishes [22, 23]. Studies suggested that chronic exposure to MPs of different size, polymers, and concentrations resulted in significant growth reduction in *Oreochromis niloticus*, *Cyprinus carpio*, and *Ambassis dussumieri* [24–26]. However, the intensity of effects varies with species, exposure duration, particle size, and type of polymer, which suggests species‐specific and parameter‐dependent responses [27, 28]. The negative effects of MPs are not only limited to growth reduction but also extend to the cellular and molecular levels of fish. MPs can significantly disrupt hematological and biochemical parameters, along with growth suppression, which are regarded as early biomarkers of toxic compound exposure in fish [22, 23]. MPs induced altered blood profiles of fish are associated with various further anomalies including immunosuppression and hematopoietic dysfunctions [29, 30]. Frequently, MPs can cause erythrocyte cellular and nuclear deformities, micronuclei formation, altered blood glucose levels, and oxidative stress [31–33]. Furthermore, they can induce reproductive dysfunctions that represent chronic threats to populations and biodiversity in addition to these abnormalities. MPs affect the reproduction system through regulating hormones and disrupting gene expression in the hypothalamic–gonadal axis, which induces histopathological damage to gonadal tissues [34–37]. Multiple studies indicated that MPs exposure reduced fecundity, gonadosomatic index (GSI), sperm motility, and hatching rate in different species like *Danio rerio*, *Clarias gariepinus,* and *Oryzias melastigma* [38–40]. In addition, there are reports on transgenerational effects that are transferred from parents to offsprings [41, 42]. MPs can induce oxidative stress through generation of excessive reactive oxygen species (ROS) and lipid peroxidation (LPO) that disrupt antioxidant defense enzymes such as superoxide dismutase (SOD), catalase (CAT), and glutathione peroxidase (GPx), and alter glutathione (GSH) levels in fish [43, 44]. Damage to cellular organelles and activation of proinflammatory pathways and suppression of anti‐inflammatory responses may also occur due to such redox imbalances that result in immune regulatory impairments [45, 46]. These immune alterations result in increased susceptibility to pathogens, reduced phagocytic activity, and histopathological damage to the fish immune system [47, 48].

Besides the physiological dysfunction, MPs exposure has been shown to disrupt normal behavioral patterns in fish. Documented abnormalities include impaired swimming performance, altered feeding behavior, disrupted predator avoidance, and abnormal reproductive behaviors [49, 50]. These disruptions are linked to oxidative stress–induced neurotoxicity, endocrine disruptions, and impaired energy metabolism, which are often accompanied by changes in neurotransmitter activity and brain histopathology [51, 52]. Such behavioral dysfunctions can reduce survivability, especially in the case of larval and juvenile stages by compromising foraging efficiency, predator avoidance, and reproductive success [53].

Although several investigations reported negative effects of MPs on specific fish species and health parameters, very few studies have covered the synthesized version of these effects across multiple physiological systems. Therefore, the objective of this review is to comprehensively analyze and synthesize the current state of research concerning the sources and transfer pathways of MPs to aquatic environment, their effects on growth performance, hematobiochemical responses, reproductive function, and behavior of fish. Since this review offers a summarized and synthesized overview of MP‐induced toxicity in fish, we hope it will be helpful in future research and policy directions concerning MPs pollution in fisheries and aquatic environments.

## 2. Methodology

**FIGURE 2:**
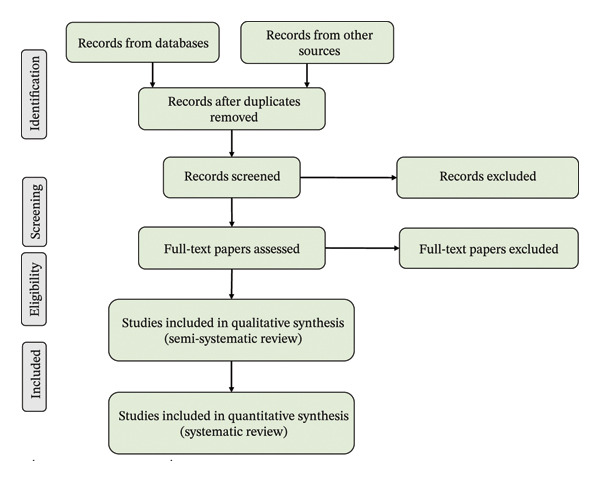
PRISMA flow diagram showing information flow at four stages of the semisystematic review process (“identification,” “screening,” “eligibility,” and “inclusion”) [54].

For a comprehensive review of MPs induced dysfunctions in fish physiology and behavior, data were collected from various literatures published from different countries around the globe. Identification of related studies was the first phase of data collection. For conducting a systematic literature search, the following specifications were created for the database. Database searched includes Google Scholar, ScienceDirect, and PubMed. Inclusion criteria: journal articles written in English, effects of MPs on fish, focusing on recent articles accessible over the internet from 2011 to 2025. To collect information, the data were searched using the keywords such as “Microplastics,” “Fish,” “Effects,” “Trophic transfer,” “Carrier effect,” “Sources,” “Pathways,” “Growth,” “Blood biomarkers,” “Hematological parameters,” “Reproduction,” “Oxidative stress,” “Immunology,” “Behavior,” “Neuro‐behavior,” “Human health,” and “Sea food,” in which “Microplastics” and “Fish” were common keywords. A total of 412 records were identified through database searching. After removal of duplicates and initial screening of titles and abstracts, 190 studies were retained for full‐text assessment. From these, studies having the predefined inclusion criteria that are relevant to the topic and availability of recent and adequate data were selected for the final analysis. Articles that failed to meet these criteria or lacked the required information were excluded (Figure 2).

**FIGURE 3:**
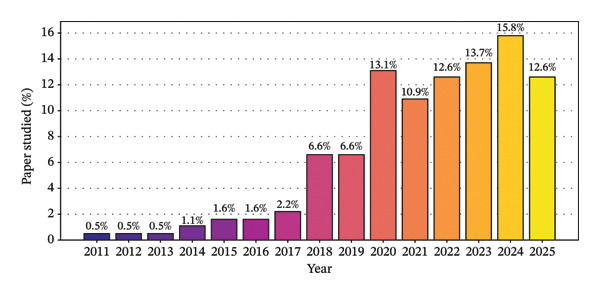
Year‐wise number of papers studied from 2011 to 2025 to synthesize the review.

Figure 3 presents the temporal distribution of studies included in this review from 2011 to 2025. The contribution of studies published during the earlier period, particularly from 2011 to 2014, was minimal, with each year accounting for less than 1.2% of the total literature. A gradual increase in the number of studies was observed between 2015 and 2017, followed by a substantial and continued rise from 2018 onward. The highest proportions of included studies were recorded in 2024 (15.8%), 2023 (13.7%), and 2020 (13.1%). Overall, the predominance of studies published from 2020 to 2025 demonstrates that this review is primarily based on the recent scientific literature, thereby ensuring that the synthesized findings are timely, relevant, and reliable.

## 3. Sources and Primary Pathways of MPs to Fish

**FIGURE 4:**
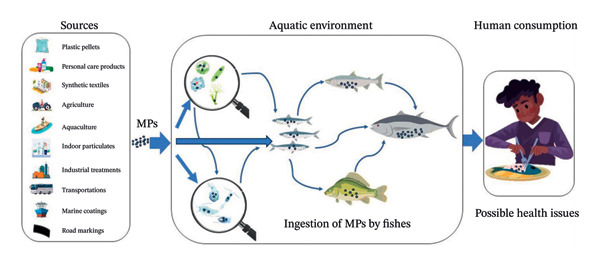
Possible trophic transfer of MPs from sources to human via aquatic organisms (figure is original and created by the authors of the manuscript).

MPs originate from two distinct types of sources. The first one is the primary sources that include microbeads, which are used in cosmetics, synthetic textile fibers, and industrial abrasives [55]. The secondary sources result from the fragmentation of larger plastic debris like packaging materials, fishing gear, agricultural plastics, and tire wear particles [55]. These materials enter aquatic systems through wastewater discharge, industrial effluents, atmospheric deposition, fragmentation of ghost fishing nets, gears, boats, etc. MPs enter fish bodythrough different pathways from the aquatic environment such as direct ingestion, trophic transfer, waterborne exposure, and sediment interactions [56] (Figure 4).

Once released into aquatic systems, MPs become available to fish through multiple pathways, depending on their feeding habits, ecological niches, and exposure routes. The most direct pathway is ingestion, where fish consume MPs by mistakes [18]. MPs can resemble any food particle, such as phytoplankton, zooplankton, algae, or detritus particles due to their variations in size, color, and shape [57]. Fish may consume these MPs mistakenly as food particles or while filter feeding leading to bioaccumulation of MPs in fish body [58]. Over time, these ingested particles can accumulate in the digestive tract and key organs and even in muscles [59]. This physical damage to intestinal linings can lead to impaired digestion, reduced nutrient absorption, and resulting poor feed conversion and growth hindrance [60].

Another significant pathway is trophic transfer, where MPs move along the food web. Small organisms such as zooplankton, benthic invertebrates, or small fish often ingest MPs, either intentionally or accidently [61]. Carnivorous fish then consume these organisms that may have previously accumulated MPs in their bodies [62, 63]. This process can result in biomagnification, where the loads of MPs and their associated chemical contaminants increase as they move up the food chain [63]. Consequently, fish at the top of the food chain accumulates higher levels of MPs and can ultimately increase the risk of being transferred to and biomagnified in humans [64]. In addition to ingestion, uptake by gills is another important entry route. Fish continuously pump large volumes of water across their gills for oxygen exchange [65]. While inhaling oxygen from water, fish may also take suspended micro‐ or nanoplastic particles through the gills [59, 66]. These particles may cause gill irritation, inflammation, and tissue damage [30].

Ingestion of benthic sediment and animals containing MPs may also contribute to this bioaccumulation process [67, 68]. Many benthic fish species feed on sediments either directly or indirectly by consuming benthic invertebrates, since sediments serve as sinks for MPs [69]; fish ingest significant amounts of sediment‐bound MPs [70]. Moreover, early life stages of fish, such as eggs and larvae, may be vulnerable to MPs exposure because of their relatively underdeveloped protective barriers and higher metabolic rates [71]. These multiple routes of exposure suggest that fish are continuously subjected to MPs contamination in aquatic environments.

Presence of MPs in fish has been widely documented across freshwater, estuarine, and marine ecosystems worldwide [72]. Several studies have reported that MPs are commonly found in the GI tract, gills, and even muscle tissues of fish [73]. The degree of the contamination depends on the location, proximity to pollution points, types of habitats, etc. [74]. However, rivers and lakes act as important channels, transporting plastic waste from land‐based sources into marine systems. The observed MPs from various aquatic ecosystems and their inhabitants come in various types including sizes (i.e., ≥ 100 nm to ≤ 5 mm in diameter), shapes (i.e., fragments, fibers, films, pellets, foams, and spheres), and colors (i.e., transparent, white, blue, black, red, yellow, and green) [75, 76]. The widespread occurrence of MPs in fish raises significant ecological and food safety concerns, as fish represent an important link between aquatic ecosystems and human consumption [77, 78].

## 4. MPs as Carrier for Cocontaminants to Fish

**FIGURE 5:**
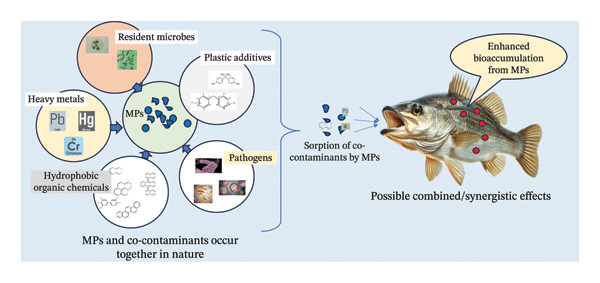
Possible co‐occurrence effects of microplastics in aquatic ecosystems (figure is original and created by the authors of the manuscript).

MPs are hazardous not only because of their own physical and chemical burden to aquatic ecosystems but also as vectors for various chemical and biological pollutants. Their physicochemical characteristics specifically high specific surface area, hydrophobicity, and porosity allow the adsorption/absorption of harmful cocontaminants present in the environment. MPs can easily sorb persistent organic pollutants (POPs), heavy metals, hydrocarbons, and pesticides, pharmaceuticals and personal care products (PPCPs), etc., on the surface of MPs, and thereby act as vectors for toxic chemicals in aquatic environments and pose the risk of enhancing bioaccumulation compared to conventional uptakes through water/food [79–81]. After being consumed by fish or other species, MPs could release these previously sorbed pollutants due to the interaction with intestinal fluids, temperature, and pH, making them viable for subsequent accumulation in body tissues and organs, where they might exert synergistic toxic effects. Therefore, the release of such chemicals within the host may result in oxidative stress, metabolic and endocrine disruptions, impaired reproduction, and compromised immunity [82, 83]. In addition, MPs may release additives such as plasticizers, stabilizers, and flame retardants that were included during the manufacture of plastics, enhancing their hazardous potential in aquatic organisms including fishes [84] (Figure 5).

In addition to chemical pollutants, MPs function as substrates for microbial colonization resulting in the formation of the so‐called “plastisphere.” This distinctive habitat would likely contain a rich microbiota that may include possibly pathogenic microorganisms such as bacteria, fungi, and viruses [85, 86]. Hence, the synergistic action of retained pollutants, leached additives, and related microbial communities makes MPs as potent carriers of multistressors. Through the vector effects, MPs facilitate the transfer of toxicants via ingestion and the trophic chain, posing significant threats to aquatic biodiversity and human health through seafood consumption [56].

## 5. MPs Induced Dysfunctions/Toxicities in Fish

MPs have emerged as a ubiquitous contaminant in aquatic environments, posing significant risks to fish health and aquatic ecosystems [87]. MPs can be readily ingested by fish due to their small size and high surface area leading to physical blockage, chemical leaching, and bioaccumulation and biomagnification. Exposure to MPs has been associated with a wide range of physiological and biochemical disturbances, including impaired growth (e.g., reduced WG, SGR and feed conversion, etc.) [26, 88, 89], hematological and biochemical alterations [90–92], reproductive disruptions (e.g., altered hormone levels, gametogenesis, GSI, etc.) [34, 39, 41], and immunological impairments (e.g., modulation of inflammatory cytokines, antioxidant enzymes, immune cell function, etc.) [45, 93, 94], and behavioral changes (e.g., altered swimming patterns and predator avoidance, etc.) [50, 52, 95]. Collectively, these dysfunctions highlight the multifaceted toxic potential of MPs, emphasizing the urgent need to understand their mechanisms of action and long‐term consequences in fish populations.

### 5.1. MPs Induced Changes in Growth of Fish

Growth performance is one of the major physiological endpoints in ecotoxicological assessments [96]. Studies have documented that exposure to MPs has adverse effects on fish growth. However, responses can be varied depending on fish species, types of polymers, particle sizes, concentrations of the exposed particles, and exposure durations. MPs ingestion can lead to damage to the GI tract, blockage in the intestine, altered feeding behavior, and nutrient absorption deficiency, eventually resulting in growth inhibition [90]. For example, *Leuresthes tenuis* exposed to low‐density polyethylene (LDPE) and high‐density polyethylene (HDPE) having a size of 75–250 μm for 14 days at over 1 × 10^3^ particles/mL showed significant reductions in growth due to both direct ingestion and trophic transfer pathways [90]. A similar symptom was also observed in *Ctenopharyngodon idella* as well [97]. Moreover, chronic exposure of *Ambassis dussumieri* to HDPE, PVC, and PS over 95 days led to severe growth impairment, demonstrating that chronic exposure increases the toxic effects over time [25]. In *Cyprinus carpio*, exposure to PVC (100–200 μm) across doses up to 136.65 μg/L inhibited WG over both 30 and 60 days significantly, highlighting the dose and time dependency of MPs toxicity [26]. Correspondingly, in *Oreochromis urolepis*, dietary ingestion of PE‐MPs resulted in damage to the intestinal structure, which led to a reduction of digestion and nutrient assimilation. These impairments promote significant growth reductions in fish [98].

Numerous studies have noted decreases in WG and SGR, including in *Neogobius melanostomus* [99] and *Oreochromis niloticus* [24, 100]. The latter study, involving MPs in diet up to 10%, also resulted in increases in feed conversion ratio (FCR), leading to reduced feed utilization. However, some studies reported insignificant or no growth effects. For example, *Clarias gariepinus* and *Symphysodon aequifasciatus* showed no significant differences in growth performance following dietary exposure to MPs, suggesting species‐specific or life‐stage‐dependent resilience [27, 28]. Similarly, *Oryzias latipes* exposed to PS particles for 150 days demonstrated no marked changes in growth performances [101]. It is to be noted that growth inhibition may not be immediate. For example, *Cyprinus carpio* exhibited normal weight during initial PS intake, but significant reductions occurred post‐MPs excretion [102]. It indicates the possible chronic effects. Overall, while some studies suggested exceptions, most of the studies report that MPs exposure results in the inhibition of normal growth processes in fish. The foremost processes include mechanical injury to the intestine, reduction in feeding efficiency, inhibition in SGR, disruption of gut microbiota, and systemic metabolic stress. MPs induced growth obstructions reported in earlier studies are summarized in Table 1.

**TABLE 1:** Summary of effects of various microplastics polymers on fish growth.

Polymer type	Size	Species	Dose	Findings	References
LDPE, HDPE	75–90 μm and 125–250 µm	*Leuresthes tenuis*	0, 1.245 × 10^3^, and 1.169 × 10^3^ particles/mL (14 days)	Direct and indirect consumption and trophic transfer reduced growth	[89]
HDPE, PVC, PS	1000 –250 µm	*Ambassis dussumieri*	0 and 0.1769 mg/L (95 days)	Chronic exposure reduced growth parameters	[25]
PVC	100 and 200 µm	*Cyprinus carpio*	0, 45.55, 91.1, and 136.65 μg/L (30, 60 days)	Inhibited weight gain and growth rate	[26]
PE	38–45 µm	*Oreochromis urolepis*	0, 1, 10, and 100 PE/mL (65 days)	Damaged small intestine–impaired digestion and nutrient absorption functions disrupted growth	[98]
PS	—	*Clarias gariepinus*	0%, 5%, 10%, and 15% of feed (30 days)	No effect on the growth performance	[27]
PE	63–75 μm/bead	*Neogobius melanostomus*	0, 1 bead/L/day (37 days)	Reduced SGR and growth performance	[99]
PS	50 and 500 µg	*Oryzias latipes*	247 and 3087 particles/L (150 days)	No effect on the overall growth performance	[101]
PE	27 and 63 µm	*Oreochromis niloticus*	4% and 8% (weight of MPs/weight of diet, w/w) (63 days)	Weight loss and decreased growth ‐decline in weight gain	[100]
PS	0.5 and 15 µm	*Ctenopharyngodon idella*	0, 100, and 500 μg/L (7 and 14 days)	Growth rate reduction on the 14^th^ day, and no change on the 7^th^ day	[97]
PS	0.10 µm	*Larimichthys crocea*	0, 5.50 × 10^−12^, 5.50 × 10^−9^, and 5.50 × 10^−7^ mg/L (14 days)	Reduction of SGR	[88]
PS	32−40 µm	*Cyprinus carpio*	0, 100, and 1000 μg/L (30 days)	Decrease in growth parameters after MPs excretion	[102]
PS	32−40 μm diameter	*Poecilia reticulata*	0, 100, and 1000 μg/L (28 days)	No effect on growth, but significant inhibition of the condition factor	[103]
PS	32–40 µm	*Symphysodon aequifasciatus*	0, 50, and 500 μg/L (30 days)	No inhibition on the growth of the fish	[28]
PS	0 μm−2000 µm	*Carassius carassius*	0, 6.38, 12.18, and 22.33 mg/fish/day (30 days)	Decline in the growth rate	[104]
LDPE	500 μm–2 mm	*Oreochromis niloticus*	0%, 2%, 4%, 6%, 8%, and 10% of diet (60 days)	Reduction of weight gain, specific growth rate, but increased FCR	[24]

*Note* *:* LDPE (low‐density polyethylene), HDPE (high‐density polyethylene), PVC (polyvinyl chloride), PS (polystyrene), PE (polyethylene), MPs (microplastics).

Abbreviations: FCR = feed conversion ratio, SGR = specific growth rate.

**FIGURE 6:**
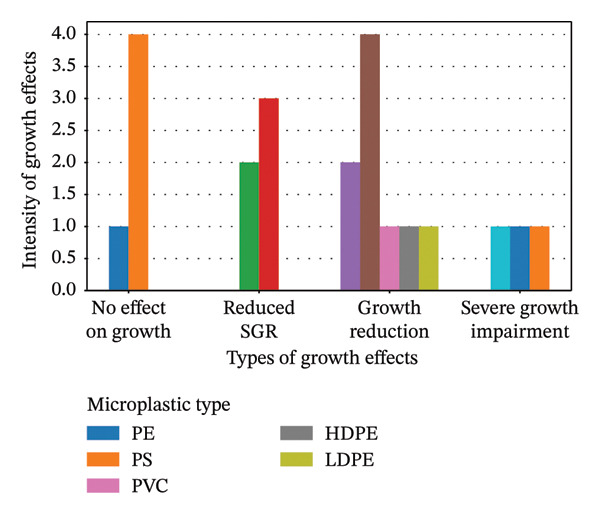
Frequency of different types of microplastics induced effects on the growth of fish.

Figure 6 compiles data from multiple studies evaluating the effects of MPs exposure on fish growth. The chart indicates that MPs significantly reduce SGR, overall growth performance, and normal growth processes. Collectively, these growth‐related impairments suggest compromised physiological condition and deteriorated health status in exposed fish.

### 5.2. MPs Induced Changes in Blood Parameters of Fish

Organisms, including fish, are very sensitive to anthropogenic pollutants like MPs [30]. To diagnose diseases and evaluate fish health, hematobiochemical parameters in fish are fundamental indices in the laboratory [29]. At the same time, they serve as sensitive bioindicators to determine the effects of different environmental contaminants on fish [31]. Once MPs enter the circulatory system, it can change the blood parameters and trigger toxicity on multiple levels including altered hemoglobin (Hb), hematocrit (Hct), mean corpuscular volume (MCV), mean corpuscular hemoglobin (MCH), mean corpuscular hemoglobin concentration (MCHC), and thrombocytes (TCs) suggesting impaired physiological and pathological states of fish [30]. MPs, along with adhered toxic chemicals, are released into the blood circulation changes immune function and gene activity in exposed fish [105]. Study found that MPs exposure caused oxidative stress, hematopoietic disruption, inflammation, and chemical toxicity, showing significantly reduced red blood cell (RBC), Hb, and Hct, and fluctuating the levels of lymphocytes, eosinophils, and neutrophils [90, 106–108]. Conversely, treatment with PS, PET, LDPE, and PA, in varying doses, significantly elevated white blood cell (WBC) and blood glucose due to activation of the immune system, inflammatory responses, or in response to oxidative damage [107, 109, 110]. But at the same time, the investigation found that *Cyprinus carpio and Etroplus suratensis,* when exposed to PE and PVC, resulted in a significant reduction of WBC in the long term, or high‐dose exposure caused a reduction of WBC because of immunosuppression, toxicity to hematopoietic tissues, and oxidative stress–induced cell death [111, 112]. MCV and MCH considerably increased when the fish were introduced to MPs at varying concentrations [107, 113, 114]. MPs can cause iron deficiency, nutritional stress, impaired erythropoiesis, and oxidative damage to RBCs, leading to increased MCV and MCH. Conversely, a sharp drop in Hb was identified, while *Oreochromis niloticus* was exposed to PA at multiple dose levels [110]. Further investigations have found that once fish are treated with MPs, it leads to decreased MCHC on account of RBC damage and Hb degradation, as well as PCV caused by RBC destruction and impaired production [107, 113, 114]. Myeloperoxidase (MPO) and malondialdehyde (MDA) level increased in several studies that elicit the active inflammation and lipid per oxidation resulting oxidative stress and cellular damage [115–117].

Exposure of MPs to fish also affects the blood urea, glucose, and cholesterol levels to a greater extent. For example, upon exposure to PE in *Pseudobagrus fulvidraco,* it triggers an increase in blood urea, glucose, and cholesterol. These results are from kidney dysfunction, liver damage, and oxidative stress in the presence of MPs [90, 92, 109, 118]. Significant alteration of plasma electrolytes was noticed whenever *Clarias gariepinus* was exposed to PE [119]. Total protein, calcium, and magnesium in the blood were considerably diminished as PE at the concentration of 400, 800, 1600, and 3200 μg/L was exposed to *Sebastes schlegeli* due to liver damage, kidney dysfunction, gill damage, disrupted homeostasis, and hormonal imbalance [120]. Overall, except some, most of the studies reported that RBC, Hb, Hct, PLT, MCHC, and PCV decreased significantly, while WBC, MCH, MCV, glucose, and total protein increased in the presence of MPs in fish blood, reflecting stress responses and immune activation. MPs induced blood toxicity in fish is summarized in Table 2.

**TABLE 2:** Summary of microplastics induced changes in blood parameters in fish.

Type	Size	Species	Doses	Increase	Decrease	References
PE	> 100 nm	*Oreochromis niloticus*	0, 1, 10, and 100 mg/L (15 days)	MCV and MCH	RBC, WBC, Hb, Hct, MCHC, platelets, and monocytes	[31]
PA	—	*Pseudobagrus fulvidraco*	0, 10, 20, 5000, and 10,000 mg/L (96 h)	SOD and CAT	Hb and Hct, GST	[32]
PE	34–50 μm	*Pseudobagrus fulvidraco*	0, 100, 200, 5000, and 10,000 mg/L (96 h)	Glucose, cholesterol, AST, ALT, ALP, SOD, CAT, and GST	RBC, Hb, Hct, calcium, magnesium, and total protein	[90]
PVC	—	*Capoeta fusca*	0, 2 mg/L (28 days)	Glucose, AST enzyme	RBC	[121]
PET, LDPE	< 5 mm	*Clarias batrachus*	0, 50 gm/kg feed (60 days)	WBC, urea and glucose, SGPT, and SGOT	RBC and Hb	[109]
LDPE	200–500 μm	*Sparus aurata*	0, 10% (90 days)	Plasma catalase, MPO, lysozyme, MDA	—	[115]
PA	125 μm–2 mm	*Pangasianodon hypophthalmus*	0, 10 mg/L (28 days)	Cellular and nuclear abnormalities, glucose	Hb	[118]
PE	22–71 μm	*Carassius carassius*	0, 4, 8, 16, 32, 64 mg/L (14 days)	SOD, CAT, GST	RBC, Hb and Hct, GSH	[108]
PS	—	*Oreochromis niloticus*	0, 0.01, 0.1, and 1 mg/0.75 g (45 days)	—	Hct	[122]
PE	34 and 125 μm	*Sebastes schlegeli*	0, 400, 800, 1600, 3200 μg/L (14 days)	Glucose, AST, ALT	RBC, Hb, protein, calcium, and magnesium	[120]
PE	—	*Clarias gariepinus*	0, 100 mg/L (15 days)	—	RBCs, thrombocytes, and lymphocytes	[119]
PE	< 0.2 mm	*Emys orbicularis*	0, 250, 500, 1000 mg/kg (28 days)	AST, ALT, cholesterol, glucose, creatinine, urea, calcium	GGT, total protein, albumin, immunoglobulin, phosphorus, LDH, globulin, and magnesium	[123]
PA	500 μm to 4 mm	*Oreochromis niloticus*	0, 10 mg/L (42 days)	Blood glucose	Hemoglobin	[110]
PE	100 nm–5 mm	*Cyprinus carpio*	0, 10 mg/L (15 days)	MCV, lymphocytes	RBC, Hb, Hct, neutrophils, WBC	[111]
PLA	69 μm	*Cirrhinus mrigala*	0%, 0.5%, 1%, 1.5%, 2%, 2.5% (90 days)	WBC, MCH, MCV	RBC, Hb, platelets, Hct, MCHC	[113]
MPs	< 100 nm	*Oreochromis niloticus*	0, 10 mg/L (70 days)	WBC, ALP, glucose, uric acid, albumin	RBC, Hb, Hct, platelets, eosinophils	[124]
PA	25–50 μm, 2 mm	*Pangasianodon hypophthalmus*	0, 10 mg/L (28 days)	Glucose	Hb	[123]
PVC	80.72 µm	*Etroplus suratensis*	0, 34.89 mg/L (10 days)	—	RBC, WBC	[112]
PET	< 5 mm	*Oreochromis niloticus*	0%, 2%, 4% (50 days)	SOD	CAT	[125]
HDPE	< 0.02 mm	*Carassius auratus*	0, 20, and 40 mg/L (28 days)	Glucose, creatinine, triglycerides, cholesterol, ALT, ALP, LDH, GGT, estradiol	HDL, LDL, total protein, albumin, globulin, testosterone	[126]
PE	< 20 µm	*Barbus sharpeyi*	0, 200, 400, 800, and 1600 mg/L (21 days)	AST, ALT, LDH and ALP, Albumin, cholesterol, total protein, triglycerides, and creatinine	—	[127]
PE	> 100 nm	*Oreochromis niloticus*	0, 10 mg/L (15 days)	Creatinine, uric acid, glucose, AST, ALT, albumin	RBC, Hb, Hct, neutrophils, and lymphocytes	[92]
PAA	0.1–0.4 mm	*Oreochromis niloticus*	0, 0.018, 0.03, and 0.09 g/L (28 days)	Total protein content	Hb, RBC, and WBC	[128]
PLA	69 µm	*Catla catla*	0%, 0.5%, 1%, 1.5%, 2%, and 2.5% (90 days)	WBC, MCH, MCV	RBC, Hb, PLT, PCV, MCHC	[114]
PS	50 nm	*Carassius auratus*	0, 1, 10, and 100 mg/kg (21 days)	CAT, SOD, GPx, MDA, ALP, ALT, AST, LDH	—	[116]
MPs	≤ 0.02 mm	*Oncorhynchus mykiss*	0, 1000, 2000 mg/kg diet (21 days)	Glucose, urea, creatinine, cholesterol, triglycerides, total protein, albumin, antioxidant enzymes: SOD, MDA, G6PDH	CAT, GPx	[117]
PLA	69 µm	*Labeo rohita*	0%, 0.5%, 1%, 1.5%, 2%, and 2.5% (90 days)	WBC, MCH, MCV	RBC, Hb, PLT, PCV, MCHC	[107]
PLA	69 µm	*Cirrhinus mrigala*	0%, 0.5%, 1%, 1.5%, 2%, and 2.5% (90 days)	WBC, MCHC, MCH, MCV	RBC, Hb, PLT, PCV	[113]

*Note:* PS (polystyrene), PE (polyethylene), PA (polyamide/nylon), PVC (polyvinyl chloride), PET (polyethylene terephthalate), LDPE (low‐density polyethylene), HDPE (high‐density polyethylene), PAA (polyacrylic acid), PLA (polylactic acid), MPs (microplastics), Hb (hemoglobin), Hct (hematocrit), PLT (platelet count), SOD (superoxide dismutase), CAT (catalase), GPx (glutathione peroxidase), MDA (malondialdehyde), G6PDH (glucose‐6‐phosphate dehydrogenase), MPO (myeloperoxidase), LDH (lactate dehydrogenase), AST (aspartate aminotransferase/SGOT), ALT (alanine aminotransferase/SGPT), ALP (alkaline phosphatase), SGPT (serum glutamate pyruvate transaminase/ALT), SGOT (serum glutamate oxaloacetate transaminase/AST).

Abbreviations: GGT = gamma‐glutamyl transferase, GST = glutathione S‐transferase, MCH = mean corpuscular hemoglobin, MCHC = mean corpuscular hemoglobin concentration, MCV = mean corpuscular volume, PCV = packed cell volume, WBC = white blood cells.

**FIGURE 7:**
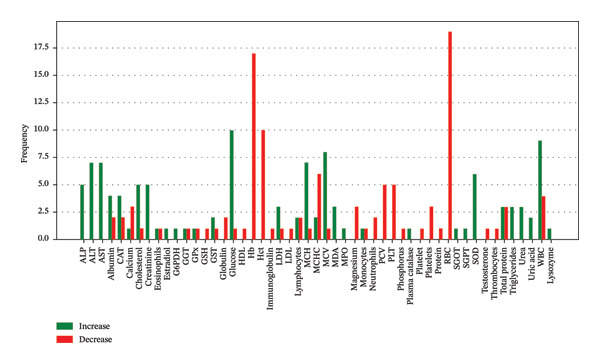
Frequency blood biomarkers reported to increase or decrease in fish exposed to microplastics.

Figure 7 analyzed data from multiple fish species documenting effects of MPs on various blood biomarkers. It shows that hematological parameters such as Hb, RBC count, and Hct are frequently reported to decrease, suggesting anemia and impaired circulatory health. In contrast, markers of oxidative stress and physiological response such as SOD, CAT, GPx, GST, MDA, glucose, and cortisol are consistently found to increase, reflecting activation of antioxidant defenses and stress pathways. Protein and lipid levels tend to remain unchanged or show inconsistent trends, indicating relative stability in these metabolic biomarkers under typical exposure conditions.

### 5.3. MPs Induced Reproductive Dysfunctions in Fish

Reproductive dysfunction caused by MPs exposure is considered one of the most critical concerns for fish health as it directly affects the population sustainability and aquatic biodiversity [129]. The reported reproductive dysfunctions by exposure to MPs include endocrine disruption, oxidative stress, histopathological damage to gonads, and alterations in gene expression related to the hypothalamic–pituitary–gonadal (*HPG*) axis [130]. The degree and nature of these alterations depend on species, polymer types, particle sizes, and concentrations of the polymers.

Several studies have reported endocrine disruption and hormonal imbalances as vital pathways affecting reproductive performances of fish. For instance, *Clarias gariepinus* exposed to LDPE (< 60 μm, 50–500 μg/L) over 96 h resulted in the suppression of gonadotropin‐releasing hormone (*GnRH*) expression [34]. In *Cyprinus carpio*, PVC (100–200 μm) at 50–150 mg/L for 60 days significantly delayed gonadal development, reduced GSI, and elevated estradiol levels, modulated HPG‐axis‐related genes (*GnRH, fsh-β, cyp19a1b or cyp19b, doublesex and Mab-3-related transcription factor 1*), and apoptosis‐regulating genes (*bax, bcl-2*) [35]. However, exposure to PS (10 μm, 2–200 μg/L) over 60 days significantly decreased GSI and hepatosomatic index (HSI) in male *Oryzias melastigma* [36]. MPs induced reduction of fecundity has also been shown in various species. For instance, *Danio rerio* showed a decline in egg production and several deformities after dietary exposure to PE after 30 days [39], and dose‐dependent loss in fecundity was observed in *Oryzias latipes* with 10 μm PS at concentrations up to 2000 μg/L over 10 weeks [40], and dietary exposure to PE and PP at 1% of feed for 5 months resulted in reduced fecundity in *Oryzias melastigma* [131].

Development of the embryo and hatching success were frequently compromised by MPs exposure. For example, *Oryzias melastigma* exposed to PE (4–6 μm, 10 mg/L for 12 days) showed disruption in the hatching success and viability of embryos [132]. Moreover, exposure to larger PS particles (600 μm, 250 mg/g for 48 h) also caused mass embryonic mortality and reduced hatching success [133]. In other studies, *Oryzias melastigma* exposed to PS (6 μm, up to 10^6^ particles/mL) exhibited accumulation of MPs on the embryonic membrane, alteration of related genes *(BMP4, NKx2.5, GATA4)* expression, incubation delay, and hatching rate reduction at different developmental stages of the egg [134]. A similar chronic exposure (2 μm, up to 200 μg/L for 150 days) resulted in the embryonic hatching success rate reduction in *Oryzias melastigma* [37].

Histological observations support testicular and ovarian damage in the number of fish species [42, 135, 136]. In *Clarias gariepinus*, PE (≥ 100 nm, 500 mg/kg for 15 days) reduced luteinizing hormone (LH) and follicle‐stimulating hormone (FSH), impaired sperm motility and viability, and caused degeneration of germinal and Leydig cells [135]. Another study on the same species with PS (197–591 μm, up to 15% in diet for 30 days) revealed tubular vacuolization and inflammation of testicular tissues [136]. Besides, in *Oreochromis niloticus*, exposure to MPs (≤ 100 nm, 10 mg/L for 15 days) resulted in the absence or reduction of spermatozoa and testicular vacuolation [124]. Beyond that, lasting reproductive effects and transgenerational toxicity are particularly a matter of concern; for example, *Paramisgurnus dabryanus* exposed to PE (8–15 μm, 1–10 mg/L) for up to 30 days results in the accumulation of MPs in gonads, induced apoptosis, and disruption of semen traits. These effects are transgenerational and have an influence on the *F1* generation, resulting in adverse effects on the development of chorionic membrane and hatchability [42]. Furthermore, *Pimephales promelas* also expressed transgenerational effects after 6 months exposure to PE (150–500 μm 100–2000 particles/L) resulting in delayed reproduction, progeny malformation, and thinner eggshells that reduce the viability and survival of embryos [41]. However, some studies report no significant effects of MPs on the reproductive organs or processes. Such findings suggested a potential resistance that might result from species‐specific or context‐dependent resilience. For instance, *Oryzi*as latipes exposed to PS (2 μm, 44 μg/L for 3 weeks) showed no prominent reproductive impacts [137]. Similarly, exposure to PE (10–300 μm, 9800 particles/g for 14 days) on *Oncorhynchus mykiss* expressed no MPs accumulation in gonads or reproductive tissues, suggesting resistance to abnormalities caused by MPs [138]. Moreover, in *Oreochromis urolepis*, MPs (38–45 μm, 0–100 particles/mL for 2–5 months) had no significant effects on fecundity or GSI, although there might be some possible impairments [139]. Other species provided further evidence of gene‐level disruptions. *Acanthopagrus latus* exposed to PE (100–2500 μm, 5 mg/L for 17 days) resulted in alterations of different genes, such as hydroxysteroid 17‐beta dehydrogenase 2 (*hsd17b2*) and estrogen receptor 2b (*esr2b*), which are involved in sex steroid metabolism and endocrine regulations in fish [140]. Moreover, *Oreochromis niloticus* exposed to 0.1–10 mg/L of MPs for 15 days experienced a significant reduction in GSI [141]; however, *Gadus morhua* exposed to PE (0.3–0.6 mm, 1% diet for 9 months) did not show any gonadal abnormalities, but alterations in the expression of vitellogenin 1 (*vtg1*) and 20β‐hydroxysteroid dehydrogenase (*20β-hsd*) were significant, indicating variations in steroidogenesis [71].

Overall, while a few studies suggested exceptions or species‐specific effects, most of the results reported that exposure to MPs led to significant impairment of normal reproductive processes in fish. However, it is clear that the underlying key mechanisms include endocrine disruption, reduction in GSI, histopathological alterations in the gonad at tissue level, reduced fecundity, hatchability, hatching rate, altered sex hormone levels, and dysregulation of HPG‐axis and reproduction‐associated gene and their expression. In some cases, these effects extend across generations as transgenerational impacts, emphasizing the long‐term risk to ecosystems and biodiversity posed by MPs in aquatic environments. The effects of MPs on different levels of reproductive performances are summarized in Table 3.

**TABLE 3:** Summary of microplastics induced reproductive dysfunctions in fish.

Type	Size	Species	Dose	Findings	References
LDPE	< 60 µm	*Clarias gariepinus*	0, 50, and 500 μg/L (96 h)	Affected *GnRH* release	[34]
PE	4–6 µm	*Oryzias melastigma*	0 and 10 mg/L (12 days)	Disrupted hatching and reduced survival of embryos	[132]
PVC	100–200 µm	*Cyprinus carpio*	0, 50, 100, and 150 mg/L (60 days)	Decreased GSI, overdue gonadal development, rise estradiol in the female, altered expression of *GnRH, gtha1, fshβ, cyp19b, erα, vtg1, dmrt1, sox9b, cyp19a*, *caspase3, bax*, *bcl-2*, *cyp19b,* and *dmrt1*genes	[35]
PE	20–27 µm	*Danio rerio*	0% and 1% of feed (30 days)	Decreased fecundity, loss ability of egg circularity, increase deformity	[39]
PS	10 µm	*Oryzias latipes*	0, 500, 1000, and 2000 μg/L (10 weeks)	Dose‐dependent reduced fecundity in mature females	[40]
PS, PP	53–100 µm	*Danio rerio*	0% and 1% of the wet weight of food (5 months)	Decreased reproductive success in offspring, premature mortality	[131]
PE, PP	53–100 µm	*Oryzias melastigma*	0% and 1% of the wet weight of food (5 months)	Reduced egg viability and quantity	[131]
PE	8–15 µm	*Paramisgurnus dabryanus*	1 and 10 mg/L (15 or 30 days)	Gonadal lesions, cell apoptosis, obstructed biological traits of semen, delayed hatching time, hatching rate, deformities, and increased mortality	[42]
PE	10–63 µm	*Oryzias melastigma*	0, 0.065, and 0.65 mg/L (12 weeks)	Reduced fecundity, lower hatching rate	[142]
PE, PP, PS	600 µm	*Oryzias melastigma*	0 and 250 mg/g (48 h)	Embryo mortality and reduced hatching rate	[133]
PS	2 µm	*Oryzias latipes*	0 and 44 μg/L (3 weeks)	No impacts on the reproduction process	[137]
PS	10 µm	*Oryzias melastigma*	0, 2, 20, and 200 μg/L (60 days)	Decreased GSI in males and females	[36]
PS	1 µm	*Danio rerio*	0, 10, 100, and 1000 μg/L (21 days)	Increased ROS in gonad; increase apoptosis testes, reduced testicular basement membrane thickness	[143]
MPs	≤ 100 nm	*Oreochromes niloticus*	0, 10 mg/L (15 days)	Reduced number of spermatozoa or no spermatozoa, vacuolated seminiferous epithelium, reduced proliferation of interstitial connective tissue	[124]
PE	≥ 100 nm	*Clarias gariepinus*	0, 500 mg/kg (15 days)	Declined LH and FSH, viable spermatocrit, sperm motility and quantity, disorganized lobule structure of tests, reduced germinal cells, interstitial cells, degeneration of germ cells, reduced spermatozoa	[135]
PS	197–591 µm	*Clarias gariepinus*	0%, 5%, 10%, 15% (30 days)	Tubular epithelial vacuolization and inflammation of testicular cells, narrowing of Sertoli cells, reduced GSI	[136]
PS	6 µm	*Oryzias melastigma*	0, 0.1, 1 × 10^3^, and 1 × 10^6^ particles/mL at 3, 6, 9, 19 dpf	Delayed incubation time, changes in heartbeat, reduced hatching rate	[134]
PS	2 µm	*Oryzias melastigma*	0, 2, 20, and 200 μg/L (150 days)	Accumulation of MPs on eggshell resulted in decreased hatching rate	[37]
PS	1 µg	*Danio rerio*	0, 10, 100, and 1000 μg/L (10 and 21 days)	Altered gonadal steroidogenic mRNA expression	[144]
MPs		*Oreochromis niloticus*	0, 0.1, 1, and 10 mg/L (15 days)	Reduced gonad weight and GSI	[141]
PE	0.3 and 0.6 mm	*Gadus morhua*	1% of feed (9 months)	Higher *vtg1* and *20β-hsd* gene expression	[71]
PE	150–500 µm	*Pimephales promelas*	0, 100, and 2000 particles/L (6 months)	Delayed reproduction, reduced egg viability, and increased malformation in offspring, thinner eggshells, delayed first clutch, malformed larvae	[41]
PE	100 μm, 500–2500	*Acanthopagrus latus*	5 mg/L (17 days)	Altered expression of *hsd17b2* and *esr2b*, disrupted endocrine regulation of reproduction.	[140]
PE	38–45 µm	*Oreochromis urolepis*	0, 1, 10, 100 MPs/mL (2–5 months)	Disrupted reproductive system	[139]
PE	10–300 μm	*Oncorhynchus mykiss*	0, 1400, and 9800 particles/g (14 days)	No evidence of reproductive tissue contamination or translocation	[138]

*Note:* LDPE (low‐density polyethylene), PE (polyethylene), PVC (polyvinyl chloride), PS (polystyrene), PP (polypropylene), MPs (microplastics), GnRH (gonadotropin‐releasing hormone), gtha1 (gonadotropin hormone alpha 1), fshβ (follicle‐stimulating hormone beta), cyp19b (cytochrome P450 aromatase b), erα (estrogen receptor alpha), vtg1 (vitellogenin 1), dmrt1 (Doublesex and Mab‐3‐related transcription factor 1), sox9b (SRY‐Box transcription factor 9b), cyp19a (cytochrome P450 Aromatase a), caspase3 (caspase‐3), bax (BCL2‐associated X protein), bcl‐2 (B‐cell lymphoma 2), hsd17b2 (17‐beta‐hydroxysteroid dehydrogenase 2), esr2b (estrogen receptor 2b), vtg1 (vitellogenin 1), 20β‐hsd (20β‐hydroxysteroid dehydrogenase), GSI (gonadosomatic index).

Abbreviations: FSH = follicle‐stimulating hormone, LH = luteinizing hormone, ROS = reactive oxygen species.

**FIGURE 8:**
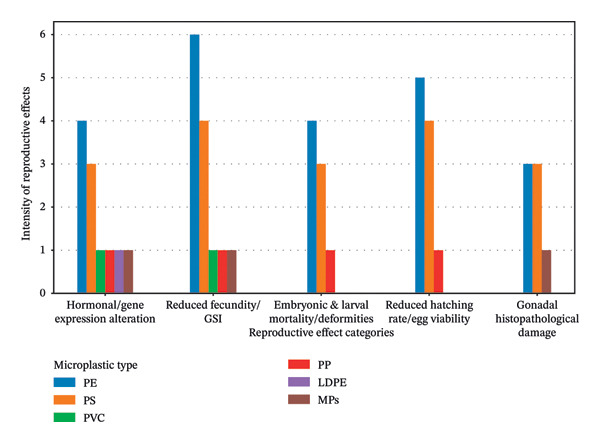
Effects of different types of microplastics on reproductive functions of fish.

Figure 8 illustrates MPs induced reproductive abnormalities in fish, including alterations in hormone levels and gene expression, reductions in GSI and fecundity, and impaired egg viability and hatching success. Additional effects encompass embryonic deformities, increased mortality during larval stages, and histopathological damage to gonadal tissues in both juvenile and adult fish. The diagram indicates that PE is associated with the majority of the reported reproductive effects.

### 5.4. MPs Induced Oxidative Stress and Immunological Dysfunction in Fish

MPs pollution is a global threat due to its persistence and effect on the environment and the organisms living in it, especially to fish altering various biological arrangements including oxidative stress and immune dysregulation [145]. Several recent studies revealed that MPs can disrupt the redox balance within fish tissues and organs depending on their types, sizes, doses, and other characteristics [146]. These types of impairments often lead to increased production of ROS, LPO, and shifts in activities of antioxidant defense enzymes such as SOD, CAT, GPx, and GSH levels. ROS and MDA levels were elevated significantly in *Carassius auratus* upon exposure to PS particles ranging from 70 nm to 50 μm at concentrations up to 1000 μg/L, representing oxidative damage [93, 94]. Similar increases of MDA level and antioxidant enzymes were detected in *Danio rerio* exposed to PS (0.1 μm and 20 μm), indicating strong proof for the dependency on the dose and duration of exposure of oxidative stress [147].

Chronic exposure to MPs displayed more prominent alterations. For example, chronic feeding of LDPE (200–500 μm) to *Sparus aurata* over 120 days led to progressive tissue impairment and antioxidant alterations that resulted from significant elevation of GPx, GSH, SOD, and MDA levels [115]. Besides, exposure to PS for 30 days at 50–500 μg/L in *Symphysodon aequifasciatus* displayed a significant elevation of SOD, CAT, GPx, and GSH activities, indicating an activated antioxidant defense system [28]. However, some studies suggested possible species‐specific tolerance or threshold effects; for example, exposure to PVC (100–200 μm) at high doses (91.1 and 136.65 μg/L) in *Cyprinus carpio* resulted in insignificant MDA or SOD responses in key organs such as the liver, gills, and intestine [26]. Besides, MPs exposure can lead to significant immunological alterations in fish. Immune dysfunction can be attributed to the activation of key regulatory pathways at the molecular level. For instance, exposure to PE in *Cyprinus carpio* activated a marked rise in the activity of genes linked to oxidative stress and cell death, including *NF-κB, IKKα, IKKβ, caspase-3*, and elements of the *NLRP3* inflammasome, suggesting that MPs can act as a catalyst in inflammatory reactions by activating key immune signaling pathways [46]. *Sparus aurata* displayed significant inflammatory responses due to dietary exposure to PS (1–20 μm), with increased expression of cytokines (*IL-1β, IL-6, and COX-2*) and suppression of anti‐inflammatory markers (*IL-10*) [45]. Recent findings further emphasize species‐specific immune responses to MPs depending on the type, dose, and particle size; for instance, in *Paramisgurnus dabryanus*, exposure to PE (1–10 mg/L for 21 days) led to significant elevation of transcript levels of intestinal immunity–related genes, serum diamine oxidase (DAO), and D‐lactic acid and induced phagocytosis and blood cell apoptosis, resulting in systemic immunity activation [48]. A similar result was observed in the case of PA exposure (25–50 μm for 28 days), causing marked erythrocytic irregularities and mucosal damage with reductions in Hb and glucose levels in *Pangasianodon hypophthalmus*, causing hematological and intestinal immune stress [142]. However, the matter of fact is that not all studies report consistent results while reporting different levels of oxidative and immune‐related biomarkers. For example, short‐term low‐dose exposures to PE (18.4 and 184 μg/L for 96 h and 50 and 250 mg/L, respectively, for 4 days) in *Pomatoschistus microps* and *Cyprinodon variegatus* did not result in significant LPO or ROS elevations at, [148, 149]. Such variations may be due to differences in particle bioavailability, metabolic rates, or resistance to the impacts caused by MPs of the particular species. Collectively, current evidence indicates that MPs are one of the most significant stressors to fish health that may negatively alter the oxidative balance and immunity. MPs induced oxidative stress and immunological dysfunction reported in earlier studies are summarized in Table 4.

**TABLE 4:** Summary of microplastic‐induced oxidative stress and immunological dysfunctions in fish.

Type	Size	Species	Doses	Findings	References
PS	70 nm, 50 µm	*Carassius auratus*	0, 10, 100, 1000 μg/L (1–7 days)	ROS, LPO, and MDA levels increased, no changes in SOD and CAT	[93]
PVC	100–200 µm	*Cyprinus carpio*	0%, 10%, 20%, and 30% of body weight (30 and 60 days)	No SOD and MDA responses in liver, intestine, and gill	[26]
PS	0.1 µm	*Oreochromis niloticus*	0, 1, 10, and 100 μg/L (1–14 days)	Insignificant MDA response and increased SOD level in liver	[150]
PET	—	*Mullus surmuletus*	Wild ingestion	GST activity increased	[151]
PS	0.1 μm, 20 µm	*Danio rerio*	0, 200 μg/L (14 days)	MDA level increased in liver, no significant change in the SOD level	[147]
LDPE	200–500 µm	*Sparus aurata*	0.5%–10% of diet (30–120 days)	GPx, GSH, SOD, and MDA levels increased	[115]
PS	70 nm, 50 µm	*Carassius auratus*	0, 10, 100, 1000 μg/L (1–7 days)	ROS level increased, mixed SOD, GPx response	[94]
PVC	0.1–1000 µm	*Carassius auratus*	0, 0.1, and 0.5 mg/L (96 h)	GPx, SOD, and MDA levels increased	[152]
PS	32–40 µm	*Poecilia reticulata*	0, 100, 1000 μg/L (28 days)	GPx, GR, GSSG, MDA, and SOD levels increased, and GSH decreased	[103]
PS	10 µm	*Oryzias melastigma*	2–200 μg/L (60 days)	GSH, MDA level increased liver, intestine, gill, testis, and organ‐specific response of SOD level	[36]
PE	1–5 µm	*Pomatoschistus microps*	0, 18.4, 184 μg/L (96 h)	No increase in the LPO level	[149]
PE	6–350 µm	*Cyprinodon variegatus*	0, 50, 250 mg/L (4 days)	Insignificant ROS level change	[148]
PVC	40–150 µm	*Sparus aurata*	0, 100, 500 mg/kg feed (15–30 days)	Increased peroxidase, immunoglobulin, and decreased phagocytosis level	[47]
PS	32–40 µm	*Symphysodon aequifasciatus*	0, 50, 500 μg/L (30 days)	Insignificant changes in lysozyme activity, increased SOD, GPx, GSH, and CAT levels	[28]
PS	70 nm, 5 µm	*Danio rerio*	0, 2, 20, and 200 μg/L (7 days)	Increased SOD and CAT	[153]
PS	5, 50 µm	*Danio rerio*	0, 100, 1000 μg/L (7 days)	Insignificant change in CAT or SOD	[154]
PE, PVC	40–150 µm	*Dicentrarchus labrax*	0, 100, 500 mg/kg (3 weeks)	No CAT or SOD response	[155]
PS	150 µm	*Danio rerio*	0, 3 mg/L (12, 24 days)	No change in SOD activity	[156]
PS	50 nm	*Oryzias melastigma*	0 and 10 μg/mL (14 days)	Increased SOD in the gut, liver	[157]
PS	50 nm, 45 µm	*Danio rerio*	0 and 1 mg/L (5 days)	No CAT response	[51]
PS	1–20 µm	*Sparus aurata*	0, 25, 250 mg/kg bodyweight/day (21 days)	Increased Lys, CSF1R, ALP, proinflammatory cytokines (IL‐1β, IL‐6, COX‐2), decreased anti‐inflammatory cytokines (IL‐10)	[45]
PS	0.25 and 8 µm	*Carassius auratus*	0, 0.05, 0.5, 5 mg/L (28 days) 300 mg/L (168 h)	Increased SOD, CAT, and HSP70 expression	[158]
PS, HDPE	Different sizes	*Danio rerio*	0, 100, 1000 μg/L (20 days)	Increased neutrophil infiltration, immune‐related gene expression alteration	[159]
PE	8 μm	*Cyprinus carpio*	1000 ng/L (21 days)	Increased NF‐κB p65, IKKα, IKKβ, p53, caspase‐3, caspase‐9, Bax, NLRP3 inflammasome, proinflammatory cytokines (TNF‐α, IFN‐γ, IL‐2, IL‐6, IL‐8, IL‐1β), decreased anti‐inflammatory cytokines (IL‐4, IL‐10) significantly	[46]
PE	8–12 µm	*Paramisgurnus dabryanus*	0, 1, 5, 10 mg/L (21 days)	Elevated serum DAO and D‐lactic acid levels, triggering immune response, phagocytosis, and blood cell apoptosis	[48]
PA	25–50 μm, 300 μm–2 mm	*Pangasianodon hypophthalmus*	0, 500 mg/kg feed	Mucosal hyperplasia, goblet cell atrophy	[123]

*Note:* PS (polystyrene), PVC (polyvinyl chloride), PET (polyethylene terephthalate), LDPE (low‐density polyethylene), PE (polyethylene), HDPE (high‐density polyethylene), PA (polyamide), SOD (superoxide dismutase) and CAT (catalase), GPx (glutathione peroxidase), GSH (glutathione), LPO (lipid peroxidation), MDA (malondialdehyde), Lys (lysozyme), ALP (alkaline phosphatase), IL‐1β (interleukin‐1 Beta), IL‐6 (interleukin‐6), COX‐2 (cyclooxygenase‐2), IL‐10 (interleukin‐10), NF‐κB p65 (nuclear factor kappa B p65), IKKα (IκB kinase alpha), IKKβ (IκB kinase beta), p53 (tumor protein p53), Bax (BCL2‐associated X protein), NLRP3 inflammasome (NLR family pyrin domain containing 3 inflammasome), TNF‐α (tumor necrosis factor alpha), IFN‐γ (interferon gamma), IL‐2 (interleukin‐2), IL‐8 (interleukin‐8), DAO (diamine oxidase), and D‐lactic acid (D‐lactic acid).

Abbreviations: CSF1R = colony stimulating factor 1 receptor, HSP70 = heat shock protein 70, ROS = reactive oxygen species.

**FIGURE 9:**
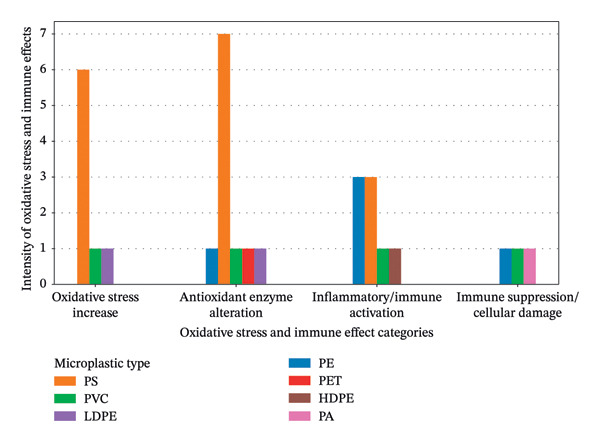
Microplastic‐induced oxidative and immunological impairments in fish.

Figure 9 illustrates oxidative and immunological dysfunctions induced by exposure to various types of MPs in fish. The chart demonstrates that MPs trigger multiple impairments associated with oxidative stress and immune imbalance. Disruption of antioxidant defense systems leads to altered activities of key antioxidant enzymes, which subsequently promote inflammatory responses and immune activation. Prolonged oxidative stress and immune dysregulation result in immunosuppression and cellular damage, thereby increasing the susceptibility of fish to intracellular and extracellular stressors. Ultimately, these pathological alterations compromise physiological homeostasis, elevate vulnerability to disease, and may contribute to increased mortality in exposed fish.

### 5.5. MPs Induced Behavioral Disruption in Fish

MPs in an aquatic environment trigger the disruption of fish behavior indirectly through physiological stress, neurological damage, and metabolic changes [49]. Several mechanisms contributing to the MPs toxicity are immune responses, oxidative stress, and disruptions of energy metabolism [51]. Oxidative stress in fish is caused by MPs, which can trigger behavioral disorders, and the toxicity of endocrine‐disrupting chemicals (EDCs) is commonly mediated through oxidative stress as a mechanism of action [160]. At the time of oxidative stress, neurons might be subject to oxidative injury and programmed cell death, accelerate the process of degeneration, and trigger inflammation of the nervous system, which alters the normal neuronal conduction and function [161]. For example, exposure of MPs to zebrafish led to an increase in exploratory behavior along with reduced aggressive and avoidance responses to predators, which was found to be significant for successful reproductive [50]. The swimming speed is significant for predation, reproduction, and self‐defense of fish, which are also affected by ingestion of MPs [49]. Studies suggest that ingestion of MPs resulted in reduced swimming speed and endurance duration, exhibiting sluggish and unstable swimming behavior in fish, as it caused physical damage, oxidative stress, and disrupted neurotransmission [94, 162–164]. For example, *Carassius Carassius* exposed to PS with different concentrations caused disorders in behavior, as well as showed defects in metabolic activities and changes in brain morphology instigated by MPs induced neurotoxicity, dysfunction in hormonal activities, and oxidative stress [52, 165]. In another research, alteration of locomotor behavior and neurobehavioral toxicity was noticed and there was a significant change in gene expression associated with the nervous system when *Danio rerio* was treated with MPs at different concentrations (10–10,000 μg/L for 6–120 h postfertilization) [53]. Another study revealed that PS (50 nm and 45 μm) induced damage in the nervous systems, neurotoxicity, and cellular damage, resulting in locomotion inhibition, notably reduced acetylcholinesterase activity in *Danio rerio* at concentrations of 1 mg/L for 3–120 h postfertilization [51]. Moreover, it has also been reported that MPs exposure caused dysfunction of the central nervous system (CNS) and unusual movements in fish [51]. Some studies revealed the elevated sedation of gonadal cell apoptosis and thinning of the testis basement membrane [143], and concurrent modulation of reproductive axis gene expression in fish when exposed to MPs resulting impaired normal reproductive behavior [34]. Furthermore, micro‐ and nanoplastics can alter the neurobehavior of fish in various interconnected pathways, affecting key neurotransmitters leading toward anxiety‐like responses, hyperactivity, and social behavior [165]. Studies revealed that MPs induced the secretion of trypsin and chymotrypsin at an increased rate, resulting in digestive hyperactivation and gut tissue disturbance, which disrupt energy allocation, gut–brain signaling, and inflammatory balance. These resulted in the alteration of swimming performance, feeding activity, and stress‐related behavioral responses in fish [166]. MPs induced oxidative stress and neuroinflammation can cause cellular damage in brain, alter gut–brain axis by dysbiosis, systemic inflammation, and altered neural function. Besides, MPs can cause metabolic and endocrine disruption by accumulating in the key organs such as liver, kidney, gut, etc., that disrupt energy balance, hormone signaling, and stress responses. These pathways combinedly disrupt the CNS and produce broad neurobehavioral effects in fish. [166]. Finally, most of the research found MPs as physical and chemical stressors that disrupt multiple physiological systems and behavioral abnormalities, including reduced swimming velocity, movements, aggression, appetite, social interactions along with increased exploratory behavior, oxygen consumption, and also leads to abnormal motor behavior, and neurobehavioral toxicity, gonadal cell apoptosis, and changes in feeding patterns. MPs induced behavioral abnormalities reported in earlier studies are summarized in Table 5.

**TABLE 5:** Summary of microplastic‐induced behavioral abnormalities in fish.

Type	Size	Species	Dose	Findings	References
PS	∼70 nm	*Danio rerio*	0, 0.5, and 1.5 mg/L (7 days)	Increased exploratory behavior, reduced aggression, and predator avoidance behavior	[50]
PS	15 µm	*Sebastes schlegelii*	0 and 190 μg/L	Reduced velocity of swimming and movement, oxygen consumption enhanced, and increased the amount of ammonia excretion	[163]
PS	24 and 27 nm	*Carassius carassius*	0.01% of BW (61 days)	Loss of appetite and changes in social behavior, metabolic defects, appearance of brain changes	[52]
PS	50 and 200 nm	*Danio rerio*	0, 10, and 1000 μg/L (6–120 hpf)	Changed in gene expression responsible for nervous system function, abnormal motor behavior, and neurobehavioral toxicity	[53]
PS	50 nm and 45 µm	*Danio rerio*	1 mg/L (3–120 hpf)	Locomotion activities are inhibited; activity of acetylcholinesterase is reduced.	[51]
PS	52 nm	*Carassius carassius*	Trophic transfer	Changes in the time of feeding, the morphology of the brain changes	[165]
PS	1 µm	*Danio rerio*	0, 10, 100, and 1000 μg/L (21 days)	Sedation, increased gonadal cell apoptosis, and thinning of the testis basement membrane	[143]
FRPM	1–5 µm	*Dicentrarchus labrax*	0, 0.26–0.69 mg/L (96 h)	Reduced swimming speed, abnormal swimming behavior, and lethargy	[94]
PS	1 nm	*Danio rerio*	50 mg/L (3–120 hpf)	Dysfunction of the central nervous system	[51]
PVC	0.1–1000 µm	*Barbodes gonionotus*	0, 0.2, 0.5, and 1.0 mg/L (96 h)	Increased trypsin and chymotrypsin activities	[166]
PS	0.5 and 15 µm	*Sebastes schlegelii*	0 and 190 μg/L (21 days)	Reduced velocity of swimming and movements	[164]
PS	1–25 μm and 500 nm	*Danio rerio*	0, 500, 5000, and 50,000 particles/mL and 0.69 mg/L (28 days)	Significant increase of seizure‐like behavior	[167]
PE	8–12 μm	*Cyprinus carpio*	0, 4.5 mg/L (60 days)	Decreased average swimming speed	[162]

*Note:* PS (polystyrene), PVC (polyvinyl chloride).

Abbreviation: FRPM = fluorescent red polymer microsphere.

**FIGURE 10:**
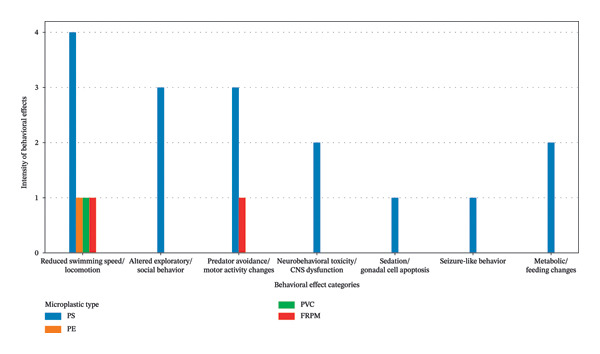
Microplastics induced behavioral alteration in fish.

Figure 10 summarizes MPs induced behavioral impairments in fish across different behavioral categories. The chart demonstrates that exposure to MPs leads to reduced swimming speed and locomotion, altered exploratory and social behavior, impaired predator avoidance and motor activity, and neurobehavioral toxicity associated with CNS dysfunction. Additional effects include sedation‐related responses, seizure‐like behavior, and metabolic or feeding alterations. Among the polymer types, PS shows the highest intensity and broadest range of behavioral disturbances, while PE, PVC, and fiber‐reinforced polymers exhibit comparatively fewer but notable effects. Overall, these behavioral disruptions indicate neurophysiological stress and compromised ecological fitness in fish exposed to MPs.

### 5.6. Variability in Experimental Design and Data Comparability

MPs toxicity data are influenced by differences in several factors. These factors include experimental design, exposure pathways, particle parameters, and doses applied. Exposure methods encompass waterborne, dietary, and trophic transfer via contaminated prey, which vary widely among the studies. The variation in exposure routes significantly affects the internal distribution, bioavailability, as well as kinetics of MPs in the fish body, leading to alterations in physiological and behavioral outcomes [166]. Generally, dietary exposure increases GI accumulation and trophic magnification [40], while waterborne exposure targets gill and integumentary uptake, accumulating and altering hematological parameters in the bloodstream [168], thereby resulting in differences in toxicity profiles.

It is to be noted that toxicological responses of MPs also vary according to their physicochemical properties, which include polymer types, particle sizes, surface charge, and so on. Multiple studies have revealed that smaller particles, such as nanosized and smaller MPs, exhibit significant enhancement of cellular internalization, induced oxidative stress, and notable potential for neurotoxicity compared to larger MPs [166]. However, some studies revealed that irregularly shaped particles exhibit higher toxicity compared with pristine spherical particles due to enhanced contaminant adsorption and surface reactivity [169]. In laboratory‐based experiments, there is a significant difference between exposure concentrations and those found in the environment [170]. Across various studies, exposure concentrations far exceed environmentally realistic concentrations to compensate for quantifiable biological effects within a limited exposure duration [170]. This approach is informative for mechanistic insights. However, it might exaggerate ecological risk when applied to natural systems. As a result, exposure concentration variability, duration, and renewal regimes evidently add to inconsistencies in reported dose–response relationships across different studies.

**FIGURE 11:**
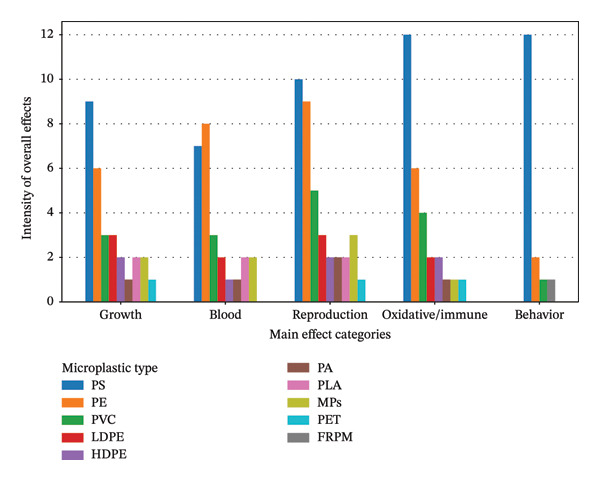
Comparative intensity of biological effects induced by different microplastic types.

Figure 11 presents a comparative assessment of the intensity of biological effects associated with different MPs polymers, including PS, PE, PVC, LDPE, HDPE, PA, PLA, PET, fiber‐reinforced plastic material (FRPM), across five major categories of effects: growth, blood‐related parameters, reproduction, oxidative and immune responses, and behavior. PS shows the highest overall intensity in most categories, with particularly strong effects on oxidative and immune responses and behavioral endpoints. PE and PVC exhibit moderate intensities, especially in growth and reproductive parameters. The remaining polymers display lower and more variable intensities of effect across various categories. The figure therefore highlights polymer‐specific differences in biological responses, indicating that the magnitude and type of effects depend strongly on microplastic composition.

## 6. Risk of MPs Contaminated Seafood Consumption in Humans

MPs are ubiquitous in the environment and pose a potential threat to human health, as reported by the World Health Organization (WHO) [171]. MPs in contaminated seafood have become a rising concern for food security all over the world and reportedly contaminate the entire marine ecosystems, including the food web and associated organisms distributed among trophic levels such as mollusks, mammals, crustaceans, and fish [172]. MPs are proven carriers of hazardous substances to organisms once ingested because their physicochemical properties facilitate the adsorption of pollutants to the particle surface [173]. Over the past few years, seafood consumption has increased intensely around the world. Concurrently, it introduces food‐borne health risks because marine organisms serve as sinks for MPs along with their resident pathogenic microbial community [174]. MPs concentrate and distribute organic components to organisms in aquatic ecosystems, which can pose a risk to wildlife and human through trophic transfer and repeated exposures [175]. MPs can also carry pollutants, pesticides, hazardous chemicals, and bioactive compounds, for example, endocrine disrupting compounds that can introduce threats to animal and human health [176].

The ingestion of MPs by humans can be caused by consuming seafood products [177]. MPs can bioaccumulate along the food chains from prey to predator [178]. Small organisms ingest MPs, and thus MPs enter the marine food web, as these ascend the food chain, consumed by predators, including fish and shellfish that humans consume, and thus humans are exposed to MPs [179]. A study found that a larger portion of fish and shellfish marketed to be consumed by humans contain MPs contamination, resulting in the dietary transfer of MPs to the human body [180]. In the environment, MPs may be accumulated in living organisms; consequently, biomagnification through trophic transfers hazardous effects to humans [181]. Humans become susceptible to allergic reactions, cell damage, and immune reactions by ingestion of these MPs [182]. Although MPs exhibit no immediate effects on human health but continuous exposure over time may result in a severe threat to human well‐being [179], chronic exposure may cause serious health issues such as dermal irritation, pulmonary complications, cardiovascular diseases, GI disorders, reproductive dysfunction, and even malignancies [173]. However, another research reported that MPs ingested by humans caused inflammatory response, endocrine disruption, neurotoxicity, bone tissue inflammation, and cytotoxicity [183].

## 7. Research Limitations

Although the experimental evidence demonstrates the adverse effects of MPs on fish physiology and behavior, however, the overall strength of this evidence remains inconsistent due to substantial methodological heterogeneity among studies. It is clear that the differences in exposure routes (dietary vs water), particle sizes, classes (pristine vs weathered), polymer types, concentrations, and exposure durations significantly limit direct cross‐study comparisons and weaken the consistent dose–response relationships. Although the studies provide significant insight from controlled laboratory experiments, there is a lack of direct use of these findings to real‐world ecological scenarios due to several limitations. For instances, in laboratories, studies are typically conducted with selected polymer types, uniform particles, and controlled exposure conditions [184]. However, natural environments are relatively unstable and contain complex mixtures of MPs with diverse sizes, shapes, aging states, and chemical additives. Moreover, environmental MPs often interact with biofilms, environmental organic matters including polychlorinated biphenyls (PCBs), polycyclic aromatic hydrocarbons (PAHs), antibiotics, and heavy metals, which can enhance their bioavailability and toxicity to fishes [185]. These types of exposures are often neglected or effects are difficult to replicate under laboratory conditions [170]. Another critical limitation is the absence of multiple ecological stressors in most experimental designs. In natural systems, fish are simultaneously exposed to both environmental stressors like temperature fluctuations, hypoxic conditions, parasite and pathogen attacks, and anthropogenic stressors like organic and inorganic chemical pollutants, changes in different water, and sediment parameters (i.e., pH, dissolved oxygen, ammonia, etc.), which may interact synergistically with MPs toxicity [186]. Furthermore, there is a time limitation in laboratory studies, as these are often conducted over short exposure durations and focus primarily on individual‐level endpoints, whereas population‐level consequences such as alteration of recruitment, trophic transfer, and ecological stability remain largely unexplored. Therefore, future research should prioritize realistic exposure scenarios relevant to the environment, long‐term multigenerational studies, and ecosystem‐based approaches to improve ecological relevance and risk assessment reliability.

## 8. Conclusion

MPs are considered proven stressors that interfere with several physiological functions of fish body, including growth, blood parameters, reproduction, immunity, and behavior, posing a significant risk to fish health. Existing research reports detrimental effects, including reduced growth, hematological alterations, hormonal dysregulation, oxidative stress, and altered behaviors. Although some species exhibit tolerance, collective evidence demonstrates that chronic exposure to MPs substantially impacts fish health, aquatic ecosystems, and eventually risks human health. These findings underscore the urgency of reducing plastic pollution and implementing strategies to monitor and reduce chances of MPs exposure in aquatic environments. To mitigate these risks, plastic inputs must be reduced, and robust management approaches should be adopted at policy levels.

## Funding

We gratefully acknowledge the financial support provided by the Bangladesh Agricultural University Research System (BAURES; Project No. 2025/18/BAU).

## Conflicts of Interest

The authors declare no conflicts of interest.

## Data Availability

The data that support the findings of this study are available on request from the corresponding author. The data are not publicly available due to privacy or ethical restrictions.
